# Novel method for rapid fluorescence ***in-situ*** hybridization of ALK rearrangement using non-contact alternating current electric field mixing

**DOI:** 10.1038/s41598-017-15515-1

**Published:** 2017-11-08

**Authors:** Satoshi Fujishima, Kazuhiro Imai, Ryuta Nakamura, Hiroshi Nanjo, Yoshitaro Saito, Hajime Saito, Kaori Terata, Yusuke Sato, Satoru Motoyama, Yoichi Akagami, Yoshihiro Minamiya

**Affiliations:** 1 Department of Thoracic Surgery, Akita University Graduate School of Medicine, Akita, Japan; 2 Akita Industrial Technology Center, Akita, Japan; 3 Division of Clinical Pathology, Akita University Graduate School of Medicine, Akita, Japan

## Abstract

Echinoderm microtubule-associated protein-like 4 gene and anaplastic lymphoma kinase gene (EML4-ALK) rearrangement is a key driver mutation in non-small cell lung cancer (NSCLC). Although Break-Apart ALK fluorescence *in situ* hybridization (FISH) is a reliable diagnostic method for detecting ALK gene rearrangement, it is too costly and time-consuming for use as a routine screening test. Our aim was to evaluate the clinical utility of a novel rapid FISH (RaFISH) method developed to facilitate hybridization. RaFISH takes advantage of the non-contact mixing effect of an alternating current (AC) electric field. Eighty-five specimens were used from patients diagnosed with NSCLC identified immunohistochemically as ALK 0, (1/2+) or (3+). With RaFISH, the ALK test was completed within 4.5 h, as compared to 20 h needed for the standard FISH. Although RaFISH produced results more promptly, the staining and accuracy of the ALK evaluation with RaFISH was equal to the standard. We found 97.6% agreement between FISH and RaFISH based on the status of the ALK signals. These results suggest RaFISH could be used as a clinical tool to promptly determine ALK status.

## Introduction

Several key genetic alterations that drive non-small cell lung cancer (NSCLC) have been detected. These include mutation of the epidermal growth factor receptor (EGFR) gene and rearrangement of the echinoderm microtubule-associated protein-like 4 gene and anaplastic lymphoma kinase gene (EML4-ALK)^1–4^. The ALK fusion oncogene is formed by a rearrangement occurring on the chromosome 2 short arm and involves the genes encoding for ALK (2p23.2) and EML4 (2p21) or, on rare occasions, other non-EML4 genes such as KIF5B and TFG. The protein product of this fusion gene has a constitutively active ALK because the basic domain of EML4 provides a mechanism for the dimerization of the new chimeric protein^1,5^. The EML4-ALK fusion gene is one of the most desirable molecular targets for patients with ALK-rearranged NSCLC because there are several specific tyrosine kinase inhibitors (TKIs) available for treatment^1^, including Crizotinib, Ceritinib, Alectinib, Brigatinib, and Lorlatinib^6,7^. Moreover, ALK TKIs are well tolerated with rapid, durable responses in ALK-positive patients, and there is potential for ongoing benefit after initial disease progression in this population^6,8^. However, ALK gene rearrangements are present in only ∼3–5% of patients with NSCLC^1,2,9^.

It is widely recognized that the detection of ALK gene rearrangements is very important for selecting effective chemotherapy for patients with advanced and/or recurrent NSCLC. Because ALK gene rearrangement involves large chromosomal inversion and translocation, fluorescence *in situ* hybridization (FISH) has become the method of choice for detecting all forms of ALK gene rearrangement, including the rare non-EML4 fusions, and it was the assay used to detect this genetic aberration in the first clinical trials of the ALK inhibitor therapy with Crizotinib^8,10,11^. As many guidelines already recommend, Break-Apart ALK FISH is a reliable standard diagnostic method in surgical pathology because it is easily applicable to formalin-fixed, paraffin-embedded (FFPE) samples, even when the exact fusion partners are not known^5^. However, whereas ALK immunohistochemistry (IHC) is a reliable and economical screening method, and a relatively easy diagnostic tool to apply for detection of pathologic (/overexpression) proteins in FFPE^12–14^, FISH is time-consuming and requires the use of expensive probes and a special fluorescence microscopy facility. Thus several unique obstacles to cost-effective and timely screening must be overcome before FISH is introduced as a routine test.

We recently developed a rapid-IHC method that makes use of an alternating current (AC) electric field to facilitate the antigen-antibody reaction, and reported its usefulness for detection of lung cancer metastasis, central nervous system tumors, and mammalian eggs^15–17^. The device reduces the time required for IHC as well as the amount of antibody required for these analyses. With this device, we apply a high-voltage, low-frequency AC electric field to the sections. The antibody is mixed within the microdroplet as the voltage is switched on and off at specific intervals. The resultant coulomb force stirs the diluted solution on the sections, which increases the opportunity for contact. This rapid-IHC method enables rapid detection of target cells in frozen sections and can provide a surgeon with an intraoperative diagnosis within about 30 min^16^. Although the rapid-IHC method still has limitations, as it has not been tested in other organs or with other detection methods, we anticipate that this technique will be applicable in multiple settings. More importantly, we demonstrated in patients with breast cancer that rapid dual *in-situ* hybridization performed with an AC electric field and this device could be used to detect human epidermal growth factor receptor 2 (HER2) amplification within 6 h^18^.

The aim of the present study was to evaluate the clinical utility, reliability and sensitivity of a novel rapid FISH (RaFISH) method that promotes ALK break-apart hybridization by taking advantage of the non-contact mixing effect in microdroplets subjected to an AC electric field.

## Results

**Figure 1:**
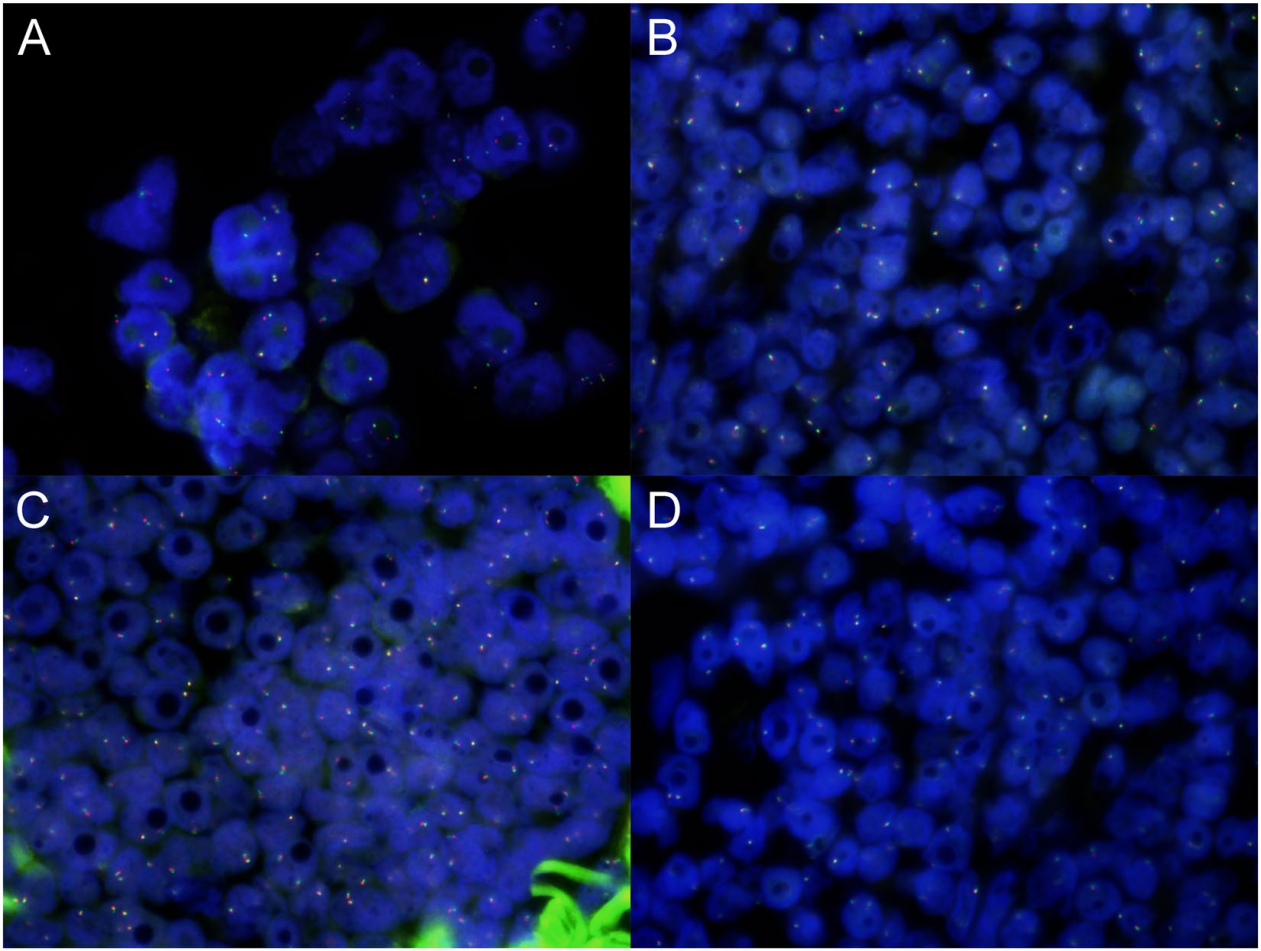
Detection of ALK rearrangements using standard Fluorescence *in Situ* Hybridization (FISH) and rapid FISH (RaFISH). Samples were classified as positive for ALK rearrangement when 15% or more of tumor nuclei showed split signals (red and green signals were separated by ≥2 signal diameters) or single red signals (3′ ALK) were observed in FISH or RaFISH. (**A**) Positive demonstrated using FISH. (**B**) Positive demonstrated using RaFISH. (**C**) Negative demonstrated using FISH. (**D**) Negative demonstrated using RaFISH.

The clinical patients’ characteristics are summarized in Table 1. For all specimens from enrolled lung cancer patients, ALK was first scored using IHC. Among the 85 specimens, 63 were scored 0, 3 were scored (1+), 0 were scored (2+) and 19 were scored (3+). Thereafter, the ALK statuses of the 85 specimens were evaluated using both FISH and RaFISH. The standard FISH using an ALK break-apart probe set and the new RaFISH protocol performed equally well (Fig. 1). On the other hand, RaFISH enabled detection of ALK break apart within 4.5 h, which is much less time than the 20 h required for standard FISH (Table 2).

**Table 1:** Patient characteristics

No. of patients		85		
Age	Male	64.9 ± 10.5	Pathological stage	
	Female	68.8 ± 10.8	IA1	6
Sex	Male	48	IA2	16
	Female	37	IA3	18
			IB	17
Tumor location			IIA	2
	RUL	24	IIB	9
	RML	6	IIIA	11
	RLL	11	IIIB	5
	LUL	24	IIIC	1
	LLL	20	IV	0
Tumor size	(mm) Mean	27.6 ± 13.4	ALK IHC status	
	Range	5–70	0	63
			1+	3
Type	Adeno	85	2+	0
			3+	19

RUL, right upper lobe; RML, right middle lobe; RLL, right lower lobe; LUL, left upper lobe; LLL, left lower lobe; adeno, adenocarcinoma.

**Table 2:** Procedural details for standard FISH and RaFISH.

Protocol	Standard	RaFISH
Dewaxing, activation, dehydration	1 h	1 h
Denaturation and hybridization	16–18 h (37 °C)	3 h (37 °C), AC
Washing slides	10 min	10 min
Staining with DAPI	10 min	10 min
Total require time	18–20 h	4.5 h

DAPI, 4′,6- Diamidino-2-phenylindole dihydrochloride; AC, alternating current electric field under the condition of 4.5 kv, 90 Hz.

Table 3 shows each ALK status defined as the ALK signal for the 85 specimens. Only two specimens in the ALK-IHC 0 group could not be evaluated using the RaFISH protocol because of poor ALK hybridization, which was caused by there being too much collagen tissue on the specimens. All 19 specimens scored 3+ using IHC were evaluated as ALK break apart signals when either FISH or RaFISH was used. We found 97.6% agreement between FISH and RaFISH based on the status of ALK signals (Cohen kappa coefficient = 0.904, 95% confidence interval (CI): 0.802–1.000).

**Table 3:** Results of ALK rearrangements based on standard FISH and RaFISH.

ALK IHC	Case	Standard		RaFISH	
0	63	Negative	63	Negative	61
		Equivocal	0	Equivocal	0
		Positive	0	Positive	0
		Not evaluable	0	Not evaluable	2
1+(/2+)	3	Negative	3	Negative	3
		Equivocal	0	Equivocal	0
		Positive	0	Positive	0
		Not evaluable	0	Not evaluable	0
3+	19	Negative	0	Negative	0
		Equivocal	0	Equivocal	0
		Positive	19	Positive	19
		Not evaluable	0	Not evaluable	0

## Discussion

In the present study, we demonstrated that RaFISH performed using our device with an AC electric field can be used to detect break-apart ALK rearrangement within 4.5 h. In addition, specimens subjected to RaFISH stained equally well and provided the same diagnosis as specimens subjected to standard FISH, and the accuracy of the evaluation using the new RaFISH technique was equal to that obtained with standard FISH.

When using FISH for detection of ALK rearrangements, the ALK Break-Apart FISH Probe Kit (Abbott Molecular, Des Plaines, IL) has become a gold standard diagnostic for targeted therapy with an ALK inhibitor in NSCLC^5^. However, FISH can be costly and time-consuming, and it requires specialized fluorescence microscopy equipment. Moreover, technical challenges include FISH signal instability and scoring difficulties. On the other hand, ALK IHC is an economical and comparatively easy screening method that is familiar and available to every pathologist and diagnostician^12–14^. Our new RaFISH system has some of the same limitations as FISH because it makes use of the ALK Break-Apart Probe set. However RaFISH has clear time advantages: ALK tests using RaFISH were completed within 4.5 h, as compared to 20 h needed for conventional FISH. Moreover, our rapid system also can be used for rapid IHC^15–17^. Prompt and correct ALK test results may save time and effort later on for many advanced and/or recurrent lung cancer patients.

There are three major conventional diagnostics for ALK fusions: highly sensitive IHC using ALK1, D5F3, 5A4, and anti-ALK, FISH including chromogenic *in situ* hybridization, and reverse-transcriptase polymerase chain reaction (RT-PCR)^12^. RT-PCR is the most sensitive and rapid method and can be used to identify specific variants of the ALK rearrangements. However, RT-PCR requires ALK fusion variants to be known so that primers for those variants can be included in the reaction. Consequently, ALK fusions with unknown partners have remained undetectable^19^. In addition, whereas IHC and FISH can be performed on a single FFPE slide, RT-PCR requires multiple slides in order to extract sufficient RNA. For these reasons, RT-PCR is not suitable for screening patients for treatment with an ALK inhibitor. This is unfortunate, as RT-PCR enables examination of specimens that are not amenable to tissue blocks, such as bronchial lavage fluid, sputum, blood, and body cavity fluids^20^. The failure rate for FISH used as a screening tool were around 4–5% in recent series^14,21^. The main disadvantage of FISH, including RaFISH, is that interpretation requires expertise and experience^22,23^. Every laboratory and hospital has to plan an appropriate molecular testing service based to the skills and resources available, and the demand and expectations placed upon it.

We first tried ALK FISH using our conventional high-voltage (4.5 KV, offset 2.4 KV), low-frequency (15 Hz) AC electric field, which we described previously^18^. However, the quality of the hybridization signal intensity for both the 3′ (telomeric) part of the fusion breakpoint and the 5′ (centromeric) part was not sufficient to stain the slide (data not shown). We speculate that this might reflect weak fluorochrome labeling on both ALK hybridization probes that came unbound with the strong shear forces caused by the AC electric mixing. We therefore changed the condition to high frequency (90 Hz) and used a polyethylene terephthalate (PET) cover to prevent probe evaporation during mixing, instead of an oil cover. Under a high-voltage (4.5 KV, offset 2.4 KV), high-frequency (90 Hz) AC electric field, the microdroplet’s shape was not transformed as the voltage was switched on and off at specific intervals. Nonetheless, in control experiments using ferrite particles (average diameter: 50 nm) in PBS, we confirmed that the particles were mixed well within the microdroplets (Supplementary Video 1). ISH requires a warm temperature, such as 37 °C, and a longer processing time than the rapid-IHC method. Consequently, evaporation can be a problem during ISH. We have shown that PET cover cap prevented evaporation when using the RaFISH device in the present study. Further investigation will be needed to more precisely define the utility of RaFISH with other kinds of ALK probes and other types of malignant cells (e.g., anaplastic large cell lymphoma and inflammatory myofibroblastic tumors).

It is also important to consider the effect of electroporation by the high-voltage (4.5 KV, offset 2.4 KV), high frequency (90 Hz) electric field when using the RaFISH technique. Pores are created when an electric current is applied to the cell membrane, which raises the possibility of false positives and over-diagnosis. In our earlier reports, we showed that the electric current does not flow through the inside of the microdroplet during AC electric field mixing^17,18^. This is because the vibrated microdroplet is separated from the electrode by both glass and air, which act as insulators.

In summary, we have shown that RaFISH can be used as a clinical tool for prompt determination of ALK status in lung cancer samples. Using RaFISH with a high-voltage, high-frequency AC electric field, break-apart ALK tests can be completed within 4.5 h, as compared to 20 h needed for conventional FISH.

## Methods

### Patients and Specimens

All experimental protocols were approved by the institutional review board at Akita University Hospital (Permit number: 1408), and written informed consent for use of samples was obtained from all patients at each hospital. The methods in this study were carried out in accordance with the approved guidelines. Nineteen EML4-ALK-positive samples for IHC and FISH were collected at the Akita University Hospital, Akita Red Cross Hospital, Omagari Kousei Medical Center, and Nakadori General Hospital between April 2008 and December 2016. Sixty-six negative/1+(2+) samples were collected for the study between January 2015 and December 2016 at Akita University Hospital. All patients enrolled in this study had undergone radical surgery for NSCLC and were analyzed retrospectively. None of the patients received preoperative chemotherapy or radiotherapy. The patients’ characteristics are listed in Table 1. Disease stages were determined according to UICC TNM classification^24^.

### Standard Immunohistochemistry

Anti-ALK IHC was performed using the iAEP method (ALK Detection Kit, Nichirei Bioscience, Tokyo, Japan) with an automatic staining protocol^13,25^. To confirm ALK positivity, IHC (and/or FISH) was also performed and evaluated by a commercial clinical laboratory (SRL, Tokyo). ALK IHC results were classified using iScore for iAEP IHC^14,26^.

### Standard fluorescence *in situ* hybridization (FISH)

Standard FISH was performed on unstained 4-μm 10% formalin-fixed, paraffin-embedded tumor tissue sections using an ALK break-apart probe set (Vysis ALK Break Apart FISH Probe kit; Abbott Molecular Inc., IL, USA) with a paraffin pretreatment reagent kit (Vysis Paraffin Pretreatment IV & Post-Hybridization Wash Buffer kit; Abbott Molecular Inc., IL, USA). Assays were performed following the manufacturer’s instructions.

### New rapid fluorescence *in situ* hybridization (RaFISH)

**Figure 2:**
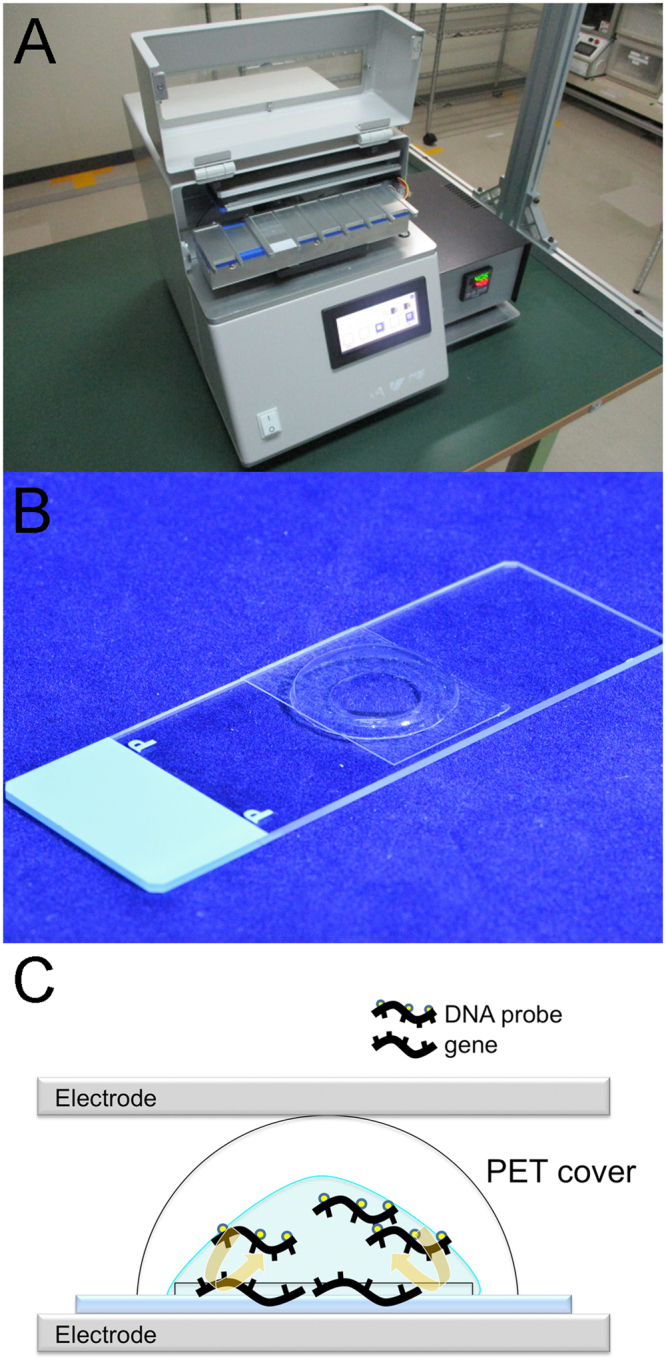
Device used to apply a high voltage, high frequency AC electric field (4.5 kv, 90 Hz). The DNA probes are mixed within microdroplets as the voltage is switched on and off. The resultant coulomb force stirs the probe solution on the sections, which increases the opportunity for contact between the probe and gene. Note that although the microdroplets’ shape is not transformed, the solution is stirred in Supplementary Video 1. (**A**) The device for Rapid Fluorescence *in Situ* Hybridization (RaFISH). (**B**) Polyethylene terephthalate (PET) cover on the slide for inhibiting evaporation of the probe solution. (**C**) Schema of the stir within a microdroplet as the voltage is switched on and off.

We used the prototype RaFISH, which we described in an earlier paper^18^, after mounting using a temperature control unit (Fig. 2A). The theory behind and technique for AC electric field mixing was described in detail previously^15–17^. Ten µl of Vysis LSI ALK Dual Color Break Apart FISH Probe and 40 µl of sterile distilled water were applied. Thereafter, a polyethylene terephthalate (PET) cover (Fig. 2B) was applied to the slide to prevent probe evaporation during mixing (Fig. 2C). The slide was placed between the electrodes, and a high-voltage (4.5 kV, offset 2.4 kV), high-frequency (90 Hz) AC current was applied after denaturation for 3 min at 73 °C. There was a distance of 3.8–4.0 mm between the slide and electrode plates, and the current was applied for 3 h at 37 °C. After washing the slide, 10 μL of DAPI counterstain were applied. Signals were analyzed using a Olympus BX51 fluorescence microscope (Olympus, Tokyo, Japan). A minimum of 50 nuclei from two separate areas of the tumor were independently scored by two technologists. Table 2 summarizes each procedure and the time required for conventional FISH and RaFISH.

### Statistical analysis

Statistical analysis was performed using JMP IN 10.0.2 software (SAS Institute, Cary, NC, USA). Cohen’s kappa-coefficient was used to assess agreement of 4 × 2 contingency tables between protocols. Values of kappa < 0.4 indicated fair-to-poor agreement, 0.4-0.8 indicated moderate-to-good agreement, and > 0.8 indicated excellent agreement.

## Electronic supplementary material

Supplementary Video 1

Supplementary Video Legend
