# Supplementary material for: Novel method for rapid fluorescence in-situ hybridization of ALK rearrangement using non-contact alternating current electric field mixing
Source: Sci Rep. 2017 Nov 8;7:15116. doi: 10.1038/s41598-017-15515-1 (PMC5678187; doi:10.1038/s41598-017-15515-1)
Supplement: Supplementary file 2 — Supplementary Video Legend [file 41598_2017_15515_MOESM2_ESM.doc]

**Novel method for rapid fluorescence in-situ hybridization of ALK rearrangement using non-contact alternating current electric field mixing**

Satoshi Fujishima^1^, Kazuhiro Imai^1^, Ryuta Nakamura^2^, Hiroshi Nanjo^3^, Yoshitaro Saito^1^, Hajime Saito^1^, Kaori Terata^1^, Yusuke Sato^1^, Satoru Motoyama^1^, Yoichi Akagami^2^,

and Yoshihiro Minamiya^1^

^1^ Department of Thoracic Surgery, Akita University Graduate School of Medicine

^2^ Akita Industrial Technology Center, Akita, Japan

^3^ Division of Clinical Pathology, Akita University Graduate School of Medicine

**Correspondence:** Kazuhiro Imai, MD PhD

Department of Thoracic Surgery, Akita University Graduate School of Medicine,

1-1-1 Hondo, Akita, 010-8543, Japan

Phone +81 18 884 6132, Fax +81 18 836 2615

Mail: [i-karo@mui.biglobe.ne.jp](mailto:i-karo@mui.biglobe.ne.jp)

**Supplementary Video 1**

Video showing ferrite particles (average diameter: 50 nm) were mixed well within the microdroplets, alothough the microdroplet’s shape was not transformed as the voltage is switched on and off at specific intervals under a high-voltage (4.5 KV, offset 2.4 KV), high-frequency (90 Hz) alternating current electric field.
